# Trends in Early-Onset Colorectal Adenocarcinoma and Neuroendocrine Tumors Across Racial and Ethnic Groups

**DOI:** 10.3390/jcm15041316

**Published:** 2026-02-07

**Authors:** Charmi Patel, Yazan Abboud, Rohan Karkra, Imran Qureshi, Paul Gaglio, Vivek Lingiah, Ahmed Al-Khazraji, Kaveh Hajifathalian

**Affiliations:** 1 Department of Internal Medicine, Rutgers New Jersey Medical School, Newark, NJ 07103, USA; 2 Division of Digestive Diseases, Ronald Reagan UCLA Medical Center, Los Angeles, CA 90095, USA; 3 Division of Liver Disease and Transplant Hepatology, Rutgers New Jersey Medical School, Newark, NJ 07103, USA; 4 Division of Gastroenterology and Advanced Endoscopy, Rutgers New Jersey Medical School, Newark, NJ 07103, USA

**Keywords:** early-onset colorectal cancer, racial and ethnic disparities, cancer incidence, cancer mortality

## Abstract

**Background:** Early-onset colorectal cancer (EOCRC), defined as diagnosis before age 50, is increasing despite declining colorectal cancer (CRC) rates among older adults. Emerging evidence suggests widening racial and ethnic disparities. We aimed to characterize long-term EOCRC incidence and mortality trends across racial and ethnic groups in the United States with comparisons by tumor subtype. **Methods:** We conducted a population-based analysis using United States Cancer Statistics data (2001–2021) for EOCRC incidence and National Center for Health Statistics data (2000–2022) for mortality. Analyses were stratified by race/ethnicity and histology. Trends were quantified using average annual percent change (AAPC) with 95% confidence intervals (Cis). **Results:** Among 474,601 early-onset adenocarcinoma (EO-ADC) cases, incidence increased in all racial and ethnic groups except Non-Hispanic Black individuals, in whom incidence declined (AAPC = −0.35%, 95% CI −0.63 to −0.08; *p* = 0.01). The steepest incidence increases occurred among American Indian/Alaska Native (AIAN; AAPC = 3.39%, 95% CI 2.70–4.15), Hispanic (AAPC = 0.94%, 95% CI 0.61–1.30), and Asian/Pacific Islander populations (AAPC = 0.64%, 95% CI 0.37–0.95; all *p* < 0.001). EOCRC mortality increased among AIAN (AAPC = 2.67%, 95% CI 1.26–4.26; *p* = 0.001) and Hispanic populations (AAPC = 0.81%, 95% CI 0.39–1.27; *p* < 0.001), but declined among Black individuals (AAPC = −1.08%, 95% CI −1.29 to −0.77; *p* < 0.001). Neuroendocrine tumors increased more rapidly than adenocarcinomas across all groups. **Conclusions:** EOCRC incidence and mortality are rising most rapidly among AIAN and Hispanic populations. Distinct incidence trajectories of colorectal neuroendocrine tumors compared with adenocarcinomas highlight the importance of histology-specific analyses for accurate epidemiologic interpretation.

## 1. Introduction

Colorectal Cancer (CRC), a malignancy arising from cells within the colon and rectum, remains a significant public health concern. It is the third most common cancer worldwide and the second leading cause of cancer-related mortality, with an estimated 1.7 million new cases and over 900,000 deaths in 2020 [1]. These numbers are projected to nearly double within 20 years [2]. In the United States alone, more than 150,000 cases were reported in 2023 [3]. Histologically, 96% of CRC cases are adenocarcinomas, while carcinoid and neuroendocrine tumors represent a small but distinct minority [4].

Decades of research have emphasized early detection and management. The United States Preventive Services Task Force (USPSTF) and national gastroenterology societies, such as the American College of Gastroenterology (ACG), recommend routine preventive screening for all adults. These strategies have contributed to declining overall CRC incidence. However, Early-Onset Colorectal Cancer (EOCRC), generally defined as CRC diagnosed before age 50, has been rising [5]. Currently, 10% of CRC cases are early onset, and this number is expected to double by 2030 [6]. Multiple large scale registry analyses show that traditional risk models calibrated for individuals over 50 perform poorly when applied to younger adults. This concerning trend is particularly notable in certain racial and ethnic groups and is thought to be influenced by environmental, social, and dietary factors.

The incidence of EOCRC has increased across multiple racial and ethnic groups, but not uniformly. Petrick et al. reported significant increases among American Indian/Alaskan Native, Hispanic, and White populations between 2001 and 2018 [7]. Other population-based studies have similarly demonstrated differential EOCRC incidence trajectories by race and ethnicity, particularly among younger age groups approaching current screening thresholds [8,9]. In parallel, emerging evidence suggests that early-onset colorectal neuroendocrine tumors, although rare, have increased in incidence and may exhibit demographic variation by race and ethnicity [10]. Mortality trends show similar inequities. Demb et al. found higher EOCRC mortality among Native Hawaiian/Other Pacific Islander and non-Hispanic Black individuals compared to non-Hispanic White populations [11]. These disparities likely reflect multifactorial causes, including genetic susceptibility, environmental exposures, and inequities in access to care.

In addition to the overall rise in EOCRC, meaningful biological and behavioral differences are thought to exist between early-onset and late-onset disease. Younger patients seem to present with more left-sided tumors, rectal primaries, and certain molecular features, including higher rates of microsatellite instability and serrated pathway lesions. Many younger patients also lack conventional risk factors such as a family history of CRC or known hereditary cancer syndromes, indicating that the drivers of EOCRC extend beyond established genetic pathways.

Despite growing recognition of EOCRC as a major public health challenge, most prior studies have examined incidence or mortality in isolation and have not jointly evaluated long-term trends across racial and ethnic groups while accounting for tumor histology. In this study, we use the United States Cancer Statistics (USCS) and National Center for Health Statistics (NCHS) databases to identify racial and ethnic disparities in early-onset colorectal adenocarcinoma and neuroendocrine tumor incidence and mortality. Our study aims to provide a comprehensive cross-histology and cross-database evaluation of EOCRC epidemiology. By integrating incidence and mortality patterns across more than two decades with a histology-specific framework, our analysis offers a more complete assessment of EOCRC trends. This analysis will help clarify which cancer variants and populations are driving the rising EOCRC burden and inform targeted health policies and interventions.

## 2. Materials and Methods

Incidence data were obtained from the United States Cancer Statistics (USCS) database for years 2001–2021 (covering ~98% of the US population). We extracted invasive CRC cases diagnosed at age < 50 and classified them by histology into early-onset colorectal adenocarcinoma (EO-ADC) and early-onset colorectal neuroendocrine tumors (EO-NET) using ICD-O codes. Age-adjusted incidence rates (per 100,000) were computed using SEER*Stat with the 2000 US standard population. Mortality data came from the CDC’s National Center for Health Statistics (NCHS) multiple-cause-of-death files (2000–2022), capturing essentially 100% of US deaths. We identified CRC deaths at age < 50 by ICD-10 codes (C18–C20). Because national mortality files do not reliably capture tumor histology, mortality analyses reflect overall EOCRC rather than histology-specific subtypes. To address this limitation, a sensitivity analysis was performed on Surveillance, Epidemiology, and End Results (SEER) data (2004–2021, ~42% coverage) to isolate EO-ADC mortality using histology codes. Individuals were grouped as Non-Hispanic White (White), Non-Hispanic Black (Black), Hispanic (of any race), Non-Hispanic American Indian/Alaska Native (AIAN), and Non-Hispanic Asian/Pacific Islander (API). We calculated annual age-adjusted rates for each subgroup and used Joinpoint regression (NCI Joinpoint program v4.9.0.1) to estimate trends. Joinpoint fits connected linear segments to log-transformed rates, identifying inflection points via a Bayesian Information Criterion. For each segment, we report the Annual Percent Change (APC) with 95% confidence interval; for the overall period we report the Average Annual Percent Change (AAPC). Statistical significance was set at two-sided *p* < 0.05. All incidence and mortality rates were standardized to the 2000 US population. Analyses were conducted using SEER*Stat version 8.4.3 and Joinpoint software. To improve comparability across datasets, we applied consistent histology definitions, age cutoffs, and weighting procedures for both incidence and mortality analyses. A minimum case threshold was required for Joinpoint modeling to avoid unstable estimates among smaller racial and ethnic groups. We also evaluated model fit and alternative Joinpoint specifications to ensure stability of estimated inflection points over time. Because incidence and mortality datasets differ in coverage and coding practices, the sensitivity analysis using SEER data served as an internal validation step. Language and grammar editing for this manuscript was assisted by artificial intelligence (AI) tools.

## 3. Results

**Figure 1:**
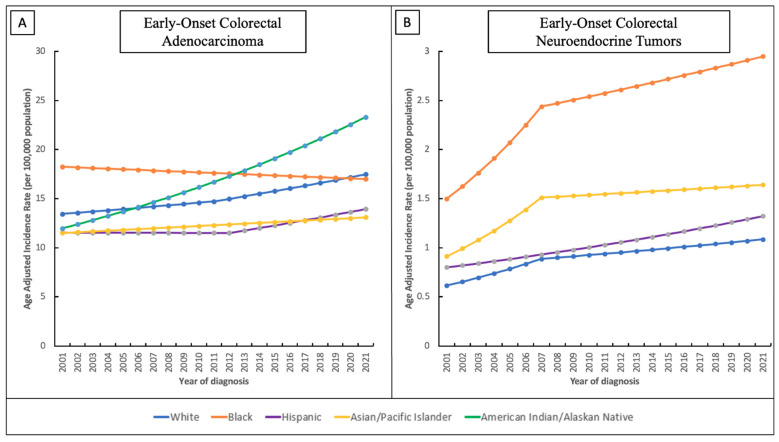
(**A**) Time-Trends and Age-Adjusted Incidence Rates Per 100,000 Population for Early-Onset Colorectal Adenocarcinoma (EO-ADC) in Different Racial/Ethnic Groups Between 2001 and 2021; (**B**) Time-Trends and Age-Adjusted Incidence Rates Per 100,000 Population for Early-Onset Colorectal Neuroendocrine Tumor (EO-NET) in Different Racial/Ethnic Groups Between 2001 and 2021.

**Figure 2:**
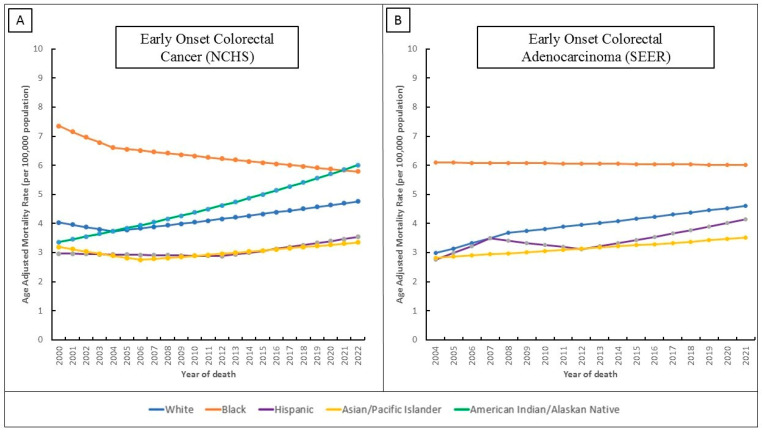
Time-Trends and Age-Adjusted Mortality Rates Per 100,000 Population in Different Racial/Ethnic Groups for: (**A**) Early-Onset Colorectal Cancer (EO-CRC) using the CDC’s National Center for Health Statistics (NCHS) Database Between 2000 and 2022 (**B**) Early-Onset Colorectal Adenocarcinoma (EO-ADC) using the Surveillance Epidemiology and End Results (SEER) Database Between 2004 and 2021.

1. EO-ADC incidence trends (2001–2021): There were 474,601 EO-ADC cases. Incidence increased over the period in nearly all groups. The Non-Hispanic White group saw a modest rise (AAPC = 1.33%, 95%CI 1.09–1.57, *p* < 0.001), with a stronger increase after 2011 (APC = 1.73%, *p* = 0.04). The Non-Hispanic Black population actually experienced a small decline in incidence (AAPC = −0.35%, 95%CI −0.63 to −0.08, *p* = 0.01). The Hispanic population had an overall slight increase (AAPC = 0.94%, *p* < 0.001), driven by a late surge after 2012 (APC = 2.14%, *p* < 0.001). API patients showed a steady rise (AAPC = 0.64%, 95%CI 0.37–0.95, *p* < 0.001). By far the largest increase was observed in AIAN individuals as their EO-ADC incidence climbed from 11.3 to 25.7 per 100,000 (2001 to 2021), corresponding to AAPC = 3.39% (95%CI 2.70–4.15, *p* < 0.001). By 2021, AIAN had the highest EO-ADC incidence of all groups (Table 1 and Figure 1).

2. EO-NET incidence trends (2001–2021): A total of 38,605 EO-NET cases were identified. Incidence increased steeply in every group. The White population saw a continuous rise (AAPC = 2.87%, 95%CI 2.22–3.73, *p* < 0.001). The Black population had an intense early increase (2001–2007 APC = 8.45%, *p* < 0.001) before leveling off (AAPC = 3.44% for 2001–2021, *p* < 0.001). The Hispanic group had a steady rise (AAPC = 2.54%, *p* < 0.001). APIs had a sharp early rise (2001–2007 APC = 8.79%, *p* < 0.001) then flattening (AAPC = 2.98% for 2001–2021, *p* = 0.007). AIAN cases were too few for reliable trend estimation (Table 1 and Figure 1).

3. EOCRC mortality trends (2000–2022, NCHS): There were 147,026 EOCRC deaths nationally. Overall mortality rates (age-adjusted per 100,000) were low (<2.0) but showed divergent trends by race. White mortality rates increased modestly (AAPC = 0.75%, 95%CI 0.55–1.00, *p* < 0.001). Hispanic mortality also rose (AAPC = 0.81%, 95%CI 0.39–1.27, *p* < 0.001). In contrast, Black mortality declined (AAPC = −1.08%, 95%CI −1.29 to −0.77, *p* < 0.001). API mortality remained essentially flat (AAPC = 0.21%, *p* = 0.55). AIAN mortality exhibited the largest rise: AAPC = 2.67% (95%CI 1.26–4.26, *p* = 0.001) over 2000–2022 (Table 2, Figure 2). AIAN, White, and Hispanic groups saw increasing EOCRC mortality, while that of the Black group improved.

4. EO-ADC mortality (2004–2021, SEER sensitivity): Focusing on the adenocarcinoma subtype (N = 46,116 deaths) yielded similar but more pronounced trends. The White group had a steep mortality increase (AAPC = 2.59%, 95%CI 2.32–2.91, *p* < 0.001), comprising a very rapid rise in 2004–2008 (APC = 5.46%, *p* < 0.001). The Hispanic group likewise had high growth (AAPC = 2.40%, *p* < 0.001) with an early surge and a later rebound (overall APCs 7.97% in 2004–2007, −2.14% in 2007–2012, +3.18% in 2012–2021). APIs showed a moderate increase (AAPC = 1.27%, *p* < 0.001). The Black group had no significant trend (AAPC = −0.08%, *p* = 0.70). AIAN deaths were too few for analysis (Detailed trends in Table 2 and Figure 2).

## 4. Discussion

In this population-based analysis spanning more than two decades, we identified substantial racial and ethnic heterogeneity in early-onset colorectal cancer (EOCRC) trends in the United States. The incidence of early-onset colorectal adenocarcinoma (EO-ADC) increased across most racial and ethnic groups, with the most pronounced rises among American Indian/Alaska Native (AIAN) and Hispanic individuals. In contrast, non-Hispanic Black individuals experienced a modest but statistically significant decline in EO-ADC incidence over the study period. Mortality trends similarly diverged by race and ethnicity, with increasing EOCRC mortality among AIAN and Hispanic populations and declining mortality among Black individuals. In parallel, colorectal neuroendocrine tumors (EO-NETs) demonstrated rapid incidence increases across multiple groups, highlighting meaningful histology-specific differences within EOCRC.

Our EO-ADC incidence findings are broadly consistent with prior U.S. registry-based studies documenting a sustained rise in EOCRC since the early 2000s. This is particularly noticed among AIAN and Hispanic populations [7,8,9]. Large population analyses have shown that EOCRC incidence is not uniformly distributed across racial and ethnic groups and that incidence trajectories have accelerated in specific populations over time [7,8]. Petrick and colleagues reported marked increases in EOCRC incidence among AIAN and Hispanic individuals between 2001 and 2018, while Chang et al. demonstrated widening racial and ethnic disparities in younger age groups [7,8]. These patterns closely mirror those observed in our EO-ADC analysis. In addition, studies examining EOCRC outcomes, including those by Zaki et al. and Tsai et al., have documented persistent racial and ethnic disparities in stage at diagnosis and survival [12,13]. This suggests that incidence trends alone do not capture the full burden of disease.

A notable and somewhat countervailing finding of our study is the favorable trend observed among non-Hispanic Black individuals. There was a demonstrated decline in EO-ADC incidence and declining overall EOCRC mortality in this population. This pattern contrasts with current literature and with the rising incidence observed in most other racial and ethnic groups, and therefore warrant careful interpretation. Importantly, this finding does not suggest elimination of colorectal cancer disparities among Black populations, who historically have borne a disproportionate burden of disease. Rather, it indicates divergence in temporal trends, which may reflect improvements in symptom recognition, earlier diagnostic evaluation, or treatment access in certain settings. Additionally, many prior studies evaluate EOCRC as a composite outcome without stratification by tumor histology. Our analysis distinguishes adenocarcinoma from neuroendocrine tumors. Given the marked early increase in EO-NET incidence followed by a levelling off in recent years in Black individuals, lack of histologic separation may partially account for these conflicting results. Lastly, interpretation of these findings must also consider known anatomic and biologic differences in colorectal cancer presentation by race. Studies have demonstrated that Black patients are more likely to present with proximal (right-sided) colon cancers compared with White patients, including among early-onset cases [14]. Proximal tumors may present with less specific symptoms and exhibit distinct molecular features, all of which have implications for diagnosis and outcomes. Accordingly, the absence of rising EO-ADC incidence among Black individuals in our study should be interpreted within the context of tumor histology specification rather than as evidence of reduced risk.

The observation that colorectal neuroendocrine tumors increased more rapidly than adenocarcinomas across racial and ethnic groups provides additional context for interpreting EOCRC trends. Although adenocarcinoma remains the dominant histology and the primary driver of screening strategies, neuroendocrine tumors represent a biologically distinct subset with different clinical behavior and diagnostic pathways. We observed disproportionately higher EO-NET incidence rates among minority individuals. Prior studies have indicated that colorectal neuroendocrine tumors are more common in Black patients than in White patients [15,16]. Reporting histology-specific trends helps avoid obscuring meaningful differences in disease epidemiology and facilitates comparison across studies that vary in case definitions.

The pronounced increases in EO-ADC incidence and mortality among AIAN and Hispanic populations likely reflect a convergence of structural, environmental, and clinical factors. These populations experience higher prevalence of metabolic risk factors such as obesity and diabetes, as well as barriers to preventive care, food insecurity, and delays in diagnostic evaluation [17,18]. AIAN communities in particular face longstanding inequities related to healthcare access and under-resourced infrastructure, which may contribute to both rising incidence and worsening outcomes. The magnitude of EO-ADC incidence increases observed among AIAN individuals represents one of the most accelerated shifts in colorectal cancer epidemiology in the United States.

While inherited cancer syndromes and germline predisposition contribute to EOCRC risk, they account for only a minority of cases [19]. Most early-onset colorectal cancer arises sporadically, which suggests an important contribution from modifiable and early-life exposures, including sedentary behavior, dietary patterns high in processed foods, obesity, and environmental factors [20]. These exposures disproportionately affect AIAN, Hispanic, and low-income communities and may help explain the incidence patterns observed in our study [17]. Evidence also suggests that differences in gut microbiome composition and inflammatory pathways may contribute to population-level variation in CRC risk, although further investigation is needed [21]

Our findings have important implications for prevention and early detection of EO-ADC. Current screening recommendations beginning at age 45 represent an important advance. However, screening uptake remains uneven across racial, ethnic, and socioeconomic groups. Younger adults frequently experience diagnostic delays when symptoms such as rectal bleeding or abdominal pain are attributed to benign conditions [22]. This is a problem that may be exacerbated in communities with limited access to primary care. Persistent disparities in screening participation and healthcare access likely contribute to the rising mortality observed among AIAN and Hispanic populations. Recent data indicate that uninsured, rural, and low-income adults aged 45–49 are significantly less likely to complete colorectal cancer screening, and healthcare infrastructure in tribal and rural areas remains under-resourced [23,24].

The rapid rise in colorectal neuroendocrine tumor incidence observed in this and other studies merits further investigation. Potential explanations include changes in diagnostic intensity, improvements in pathologic classification, and true shifts in tumor biology. Because these tumors are often diagnosed incidentally or symptomatically rather than through screening, future work should evaluate stage distribution, diagnostic pathways, and temporal changes in classification practices to better understand the drivers of these trends.

Limitations of our study include lack of detailed patient-level information on risk factors that could influence early-onset CRC incidence such as family history, smoking status, and other comorbidities. Large national databases (USCS, NCHS, SEER) may also contain missing or miscoded records, and differences in data collection across sources can introduce heterogeneity. Additionally, we did not perform stratification of colorectal cancer by anatomic sub-site, which may limit insight into proximal versus distal tumor–specific trends across racial and ethnic groups. As an observational analysis, we recognize we cannot establish causality or adjust for all unmeasured confounders. These limitations should be kept in mind when interpreting our findings.

In summary, EO-ADC incidence and mortality continue to rise most rapidly among AIAN and Hispanic populations, while non-Hispanic Black individuals demonstrate declining EO-ADC incidence and overall EOCRC mortality. Colorectal neuroendocrine tumors show distinct and rapidly increasing incidence patterns that should be interpreted as histology-specific epidemiologic trends rather than drivers of screening policy. Addressing EOCRC disparities will require targeted prevention strategies, equitable access to screening and diagnostic services, and focused efforts to reduce structural barriers in the populations most affected.

## Figures and Tables

**Table 1:** Time-Trends of Early-Onset Colorectal Adenocarcinoma (EO-ADC) and Early-Onset Neuroendocrine Tumors (EO-NET) Incidence Rates Between 2001 and 2021 Across Different Racial/Ethnic Groups.

Race/Ethnicity	Early-Onset Colorectal Cancer Cases (N = 544,760) ^a^	Trends ^b^				
		Time Period	APC (95% CI)	*p*-Value	AAPC (95% CI)	*p*-Value
Early-Onset Colorectal Adenocarcinoma (N = 474,601; 87.1%)						
White	319,525 (58.7%)	2001–2011	0.92 (−1.16 to 2.81)	0.22	1.33 * (1.09 to 1.57)	<0.001
		2011–2021	1.73 * (1.06 to 3.90)	0.04		
Black	69,555 (12.8%)	2001–2021	−0.35 * (−0.63 to −0.08)	0.01	−0.35 * (−0.63 to −0.08)	0.01
Hispanic	55,206 (10.1%)	2001–2012	−0.03 (−1.58 to 0.68)	0.89	0.94 * (0.61 to 1.30)	<0.001
		2012–2021	2.14 * (1.43 to 3.77)	<0.001		
Asian/Pacific Islander	22,276 (4.1%)	2001–2021	0.64 * (0.37 to 0.95)	<0.001	0.64 * (0.37 to 0.95)	<0.001
American Indian/Alaskan Native	4309 (0.8%)	2001–2021	3.39 * (2.70 to 4.15)	<0.001	3.39 * (2.70 to 4.15)	<0.001
Early-Onset Colorectal Neuroendocrine Tumors (N = 38,605; 7.1%)						
White	19,398 (3.6%)	2001–2007	6.27 * (3.75 to 14.37)	<0.001	2.87 * (2.22 to 3.73)	<0.001
		2007–2021	1.45 * (0.23 to 2.15)	0.03		
Black	9908 (1.8%)	2001–2007	8.45 * (4.28 to 24.79)	<0.001	3.44 * (2.42 to 4.95)	<0.001
		2007–2021	1.37 (−0.78 to 2.39)	0.1		
Hispanic	4859 (0.9%)	2001–2021	2.54 * (1.78 to 3.49)	<0.001	2.54 * (1.78 to 3.49)	<0.001
Asian/Pacific Islander	2676 (0.5%)	2001–2007	8.79 * (2.90 to 47.89)	<0.001	2.98 * (1.17 to 5.60)	0.007
		2007–2021	0.59 (−5.94 to 1.96)	0.53		
American Indian/Alaskan Native	322 (0.1%)	^				

^ There were too few cases per at least one calendar year hindering the calculation of an incidence rate and thus time-trend. ^a^ Data are presented as count numbers followed by percentages of the count numbers from the total cases of early-onset colorectal cancer in the database. ^b^ Time-trends were computed using Joinpoint Regression Program (v4.9.0.1, NCI) with 3 maximum Joinpoints allowed (4-line segments). * Implies statistical significance APC: Annual Percentage Change, AAPC: Average Annual Percentage Change.

**Table 2:** Time-Trends of Early-Onset Colorectal Cancer Mortality Rates Between 2001 and 2020 Across Different Racial/Ethnic Groups using the CDC’s National Center of Health Statistics (NCHS) Database, and Sensitivity Analysis of Early-Onset Colorectal Adenocarcinoma Cases using the Surveillance, Epidemiology, and End Results (SEER) Database.

Race/Ethnicity	Early-Onset Colorectal Cancer Deaths N (%) ^a^	Trends ^b^				
		Time Period	APC (95% CI)	*p*-Value	AAPC (95% CI)	*p*-Value
Early-Onset Colorectal Cancer (N = 147,026) using the CDC’s NCH Database						
White	96,841 (65.9%)	2000–2004	−1.95 * (−5.78 to −0.07)	0.04	0.75 * (0.55 to 1.00)	<0.001
		2004–2022	1.36 * (1.15 to 1.66)	<0.001		
Black	27,284 (18.6%)	2000–2004	−2.63 * (−5.91 to −0.95)	0.01	−1.08 * (−1.29 to −0.77)	<0.001
		2004–2022	−0.73 (−1.00 to 0.49)	0.08		
Hispanic	15,291 (10.4%)	2000–2012	−0.25 (−3.65 to 0.61)	0.54	0.81 * (0.39 to 1.27)	<0.001
		2012–2022	2.09 * (1.22 to 5.53)	0.005		
Asian/Pacific Islander	6035 (4.1%)	2000–2006	−2.49 (−13.18 to 1.49)	<0.001	0.21 (−0.50 to 1.30)	0.55
		2006–2022	1.25 (−1.54 to 6.62)	0.05		
American Indian/Alaskan Native	1258 (0.9%)	2000–2022	2.67 * (1.26 to 4.26)	0.001	2.67 * (1.26 to 4.26)	0.001
Sensitivity Analysis: Early-Onset Colorectal Adenocarcinoma (N = 46,116) using the SEER Database						
White	24,954 (54.1%)	2004–2008	5.46 * (3.81 to 8.50)	<0.001	2.59 * (2.32 to 2.91)	<0.001
		2008–2021	1.73 * (1.35 to 2.03)	<0.001		
Black	8260 (17.9%)	2004–2021	−0.08 (−0.50 to 0.36)	0.70	−0.08 (−0.50 to 0.36)	0.70
Hispanic	9036 (19.6%)	2004–2007	7.97 * (3.94 to 15.99)	0.001	2.40 * (1.98 to 3.10)	<0.001
		2007–2012	−2.14 * (−5.73 to −0.02)	0.04		
		2012–2021	3.18 * (2.37 to 4.61)	0.004		
Asian/Pacific Islander	3416 (7.4%)	2004–2021	1.27 * (0.68 to 1.94)	<0.001	1.27 * (0.68 to 1.94)	<0.001
American Indian/Alaskan Native	401 (0.9%)	^				

^ There were too few cases per at least one calendar year hindering the calculation of an incidence rate and thus time-trend. ^a^ Data are presented as death numbers followed by percentages of the death numbers from the total deaths from early-onset colorectal cancer in each database. ^b^ Time-trends were computed using Joinpoint Regression Program (v4.9.0.1, NCI) with 3 maximum Joinpoints allowed (4-line segments). * Implies statistical significance. APC: Annual Percentage Change, AAPC: Average Annual Percentage Change.

## Data Availability

Publicly available datasets were analyzed in this study. Data on early-onset colorectal cancer incidence were obtained from the United States Cancer Statistics (USCS) database (https://www.cdc.gov/cancer/uscs, (accessed on 1 January 2025), and mortality data were obtained from the National Center for Health Statistics (NCHS) Multiple Cause of Death database (https://wonder.cdc.gov/, (accessed on 1 January 2025). All data sources are publicly accessible and de-identified. No new data were created or analyzed beyond these publicly available datasets. All figures and tables in this manuscript are original and do not infringe upon any copyright.

## Abbreviations

The following abbreviations are used in this manuscript:

AAPC	Average Annual Percent Change
ADC	Adenocarcinoma
AIAN	American Indian/Alaska Native
API	Asian/Pacific Islander
CRC	Colorectal Cancer
EO-ADC	Early-Onset Adenocarcinoma
EO-NET	Early-Onset Neuroendocrine Tumor
EOCRC	Early-Onset Colorectal Cancer
NCHS	National Center for Health Statistics
NHB	Non-Hispanic Black
NHW	Non-Hispanic White
SEER	Surveillance, Epidemiology, and End Results
USCS	United States Cancer Statistics
